# Electronic referral system policy analysis: a qualitative study in the context of Iran

**DOI:** 10.1017/S1463423624000239

**Published:** 2024-10-17

**Authors:** Mohammad Javad Kabir, Alireza Heidari, Mohammad Reza Honarvar, Zahra Khatirnamani

**Affiliations:** Health Management and Social Development Research Center, Golestan University of Medical Sciences, Gorgan, Iran

**Keywords:** electronic referral system (e-Referral), e-Health, Golestan province, policymaking, policy analysis, policy triangulation framework, qualitative research

## Abstract

**Background:**

Referral system is among the key elements of primary health care that leads to enhanced efficiency, reduced costs, reduced waiting time, and patients’ enhanced access to more specialized services. The present study was aimed at analysing the policies of the electronic referral (e-Referral) system in Iran.

**Methods:**

This qualitative study was conducted based on Walt and Gilson’s policy triangle and Kingdon’s models. Data were collected through document analysis and 51 semi-structured interviews with managers at various levels, experts, family physicians, specialist physicians, and patients. Document analysis was performed by content analysis method, and interview analysis was performed through framework analysis method in Atlas.ti 8.

**Results:**

The e-Referral system was launched with the aim of equitable access to services and to benefit from better management of health resources. Valid scientific evidences were used to formulate policies. Numerous meetings were held with domestic and foreign stakeholders at the provincial, city, and rural levels. The implementation of the programme followed a bottom-up approach, and the main obstacles to the implementation of the programme included the change of senior managers of the health system and their not being fully aware of the importance of the programme, inadequate allocation of financial resources, and unwillingness of some patients to follow the referral system.

**Conclusions:**

The policy triangle framework, while explaining the components of the e-Referral system programme, revealed the obstacles to the proper implementation of the programme. In order to ensure that the programme goes on continuously and successfully, it is essential to create the necessary determination and commitment on the part of the minister of health and medical education and senior managers of the health system, earmark resources for the programme, improve resource allocation with insurance management, reform the payment system, plan to raise public awareness, and attract community participation.

## Introduction

Health is one of the basic rights of every individual in the society, and the government is obliged to provide it equally and with justice (Damari *et al.*, 2020). According to the Declaration of Alma-Alta, the key to Health for All (HFA) was primary health care (PHC) (Kabir *et al.*, 2013). A key element of PHC is the referral system, in which patients may access medical care in health centres prior to accessing higher level care, such as specialist and sub-specialist hospitals (Abrahim *et al.*, 2015). In a referral system, health care provider centres are divided into three levels. The first level is the health centres wherein family physicians are based. The second level is the specialized centres that are tasked with providing specialized services to patients referred from the first level. The third level is the sub-specialty centres that provide sub-specialty services to patients referred from the second level (Maleki Rastaghi *et al.*, 2018).

One of the main features of health care systems is that there is a relationship between the care provided at the first and second level. Physicians providing PHC as ‘gatekeepers’ have a responsibility to determine which patients need secondary care. The referral system is an organizational structure for referring medical problems from general practitioners to specialists (Akbari *et al.*, 2005) and in the referral system a health personnel, due to lack of knowledge, skills, drugs, and equipment, directs the patient to higher levels to receive specialized diagnostic and therapeutic services (Janati *et al.*, 2017). The use of referral system leads to reduction in specialized service costs and hospital services (Abbasi *et al.*, 2021; Kavian Telouri *et al.*, 2020), greater efficiency and effectiveness of the health care system, as well as creating justice and patients who need more access to more specialized services (Nasrollahpour Shirvani *et al.*, 2010) and whereas paper referrals due to incomplete information that patients provide may lead to delays in patient referrals (Hughes *et al.*, 2018), electronic referral (e-Referral) systems were designed to improve waiting time and efficiency by electronic standardization of information and communication in the referral process (Allison *et al.*, 2009).

Iran is located in the Eastern Mediterranean region with a population of more than 80 million (Alinia & Davoodi Lahijan.,^2019^). According to the Budget Law of Iran in 2005, the Health Services Insurance Organization was assigned to issue health insurance booklet and provide health services to all residents of rural and urban areas with a population of less than 20,000 people to provide health services in the form of family physicians and through the referral system (Chaman *et al.*, 2012). In line with this, paragraph (b) of Article 91 of the Law of the Fourth Economic, Social and Cultural Development Plan of the Islamic Republic of Iran (2005–2009) stated that by the end of this programme, the necessary arrangements should be made for the establishment of health insurance with a focus on family physicians and the referral system. Following the approval of the Islamic Consultative Assembly of Iran (i.e. the Iranian Parliament), the implementation of this plan began in the second late 2005 (Babazadeh Gashti *et al.*, 2016). Although, since 1985, significant achievements have been made with the implementation of the health care network system and service levelling, and despite the fact that the upstream laws of Iran underscore access to specialized services at higher levels through the referral system, appropriate referral system was not materialized (Nasrollahpour Shirvani *et al.*, 2010). Therefore, various studies have emphasized the importance and necessity of using a referral system (Tavakoli *et al.*, 2017; Maftoon *et al.*, 2016).

Designing referral systems are relatively easy, but their implementation is so difficult. The efficiency of the referral system depends on the availability of sufficient personnel, appropriate information system, ease of data transfer, and control costs (Dehnavieh *et al.*, 2017). Countries such as the United Kingdom, Finland, Denmark, Norway, the Netherlands, New Zealand, Australia, and the United States have all managed to implement national e-Referral systems. However, the deployment of these systems often takes place very slowly, and have been implemented regionally rather than nationwide (Bouamrane & Mair, 2014). The Golestan province, which is located in the north of Iran, 2014, started completing the electronic health record (EHR) process by designing health information software, and given the importance of establishing an e-Referral system and benefitting from its advantages, launched the e-Referral system as a pilot in the country (Kabir *et al.*, 2021).

Challenges for implementation of the e-Referral system include inadequate planning, shortage of workforce, scheduling, patient load and waiting time, lack of feedback from the specialist to the family physician, insufficient skilled family physicians, limited financial resources, late payments, patients’ resistance on specialist referral, lack of public awareness about e-Referral system, and lack of suitable infrastructure for communications technology infrastructure in some rural areas (Kabir *et al.*, 2023).

Whereas policy analysis may contribute to understanding the complexity of the policy process and its nature, and provide knowledge and evidences on related to policymaking regarding problems (Fischer & Miller, 2017), it also helps policymakers to improve the chances of successful implementation of prospective policies and provide opportunities to make policy documents (Yousefinezhadi *et al.*, 2017). Analysis of policies of the e-Referral system programme may also lead to the identifying of adverse consequences of the policy implementation, as well as related barriers to achieving the goals of the programme. Using the results of the analysis, policymakers can review and revise policies to meet the real needs of the people. Therefore, the purpose of this study is to analyse the policies of the e-Referral system.

## Methods

A qualitative study was conducted in 2019 using Walt and Gilson’s triangle and Kingdon’s multiple stream models in Iran. Two methods, i.e. document review and in-depth individual interviews, were used for collecting the data.

Collecting documents was performed in a purposeful manner and by referring to the e-Referral headquarters in-person, the Office of Deputy of Health and the Deputy of Treatment of Golestan University of Medical Sciences and the Health Service Insurance Organization and by searching for sources and documents, visiting websites and internal databases concentrating keywords (content of policies and programmes of e-Referral system, implementation process, contextual factors, and stakeholders) based on criteria search. Upstream documents, reports, minutes of meetings, published interviews, and news posted on the country’s internal websites were the main documents used in the study of the laws.

Semi-structured interviews were used to collect data of experts’ perspective. Participants in the study were 51 senior, middle, and operational managers of the Office of Deputy of Health, and Deputy of Treatment and Statistics and Information Technology Department of Golestan University of Medical Sciences, General Department of Health Insurance of Golestan Province, administrators of health networks in the province, family physicians, medical specialists, experts, and patients referred by family physician to specialist physician. Data collection went on until reaching data saturation and not obtaining new data anymore.

The characteristics of managers, experts, and service providers included sufficient experience and active contribution in the development, establishment, and evaluation of the e-Referral system and the tendency towards participating in the study. These characteristics were at the level of service recipients using the health services through the e-Referral system (receiving appointments from the first level of service delivery and being visited at the second level of service delivery) and the desire to express the process of receiving services and challenges they faced with. An interview guide was developed for conducting the interviews. The questions covered various aspects: the content of the policy, the context of the policy, the policymaking process, and the stakeholders involved in the e-Referral system. Each interview took a minimum of 35 minutes and a maximum of 65 minutes (average 50 minutes). Prior to the interviews, the purpose of the study was fully explained to the participants. The participants are informed that their information will be kept confidential. Note-taking was also used while recording conversations. Following performing the interviews, the verbatim of the recorded interviews were transcribed. The text of the tapes was written down as soon as possible. The participants were provided with the obtained data and they were asked to comment on accuracy of the results. Following conducting each interview, coding was performed. Lincoln and Guba criteria including dependability, transferability, credibility, and confirmability were used to improve the rigor and trustworthiness of the study (Shenton, 2004; Damari & Heidari, 2020; Damari *et al.*, 2021). The participants reviewed and confirmed the data and codes to establish credibility. To ensure dependability, the authors tried to recruit participants with maximum variance sampling. To increase dependability, the categories and sub-categories, and extracted codes were reviewed by two faculty members who were familiar with qualitative analysis and were not part of this study. For transferability, the study background and participants’ characteristics were described and the study was compared to similar researches to ensure that the results are consistent.

To analyse the data, framework analysis was used, which includes five main steps:

(1) **Familiarization:** The voices were listened to again and the manuscripts were read again and the ambiguities were removed.

(2) **Identifying a thematic framework:** The basic conceptual framework was based on a review of the literature on the dimensions of the policymaking triangle model.

(3) **Indexing:** Units or parts of the data related to a certain theme were identified, and the transcribed text was indexed using codes related to the themes and sub-themes of the conceptual framework.

(4) **Charting:** Data were summarized as thematic tables.

(5) **Mapping and interpretation:** All data were reviewed again based on a conceptual framework. A table was created for each theme. Every single row of each table represented an interviewee who was identified by the appropriate code. The columns of the table also represented themes and sub-topics. The original transcribed files were repeatedly reviewed and completed by the main researcher during the analysis period. The resulting diagram was interpreted to compare the data obtained for individuals and sub-themes, and the relationship between themes and sub-themes was investigated.

Thematic content analysis was used to analyse the documents, and to supplement and validate the findings of the interviews. Atlas.ti 8 was used to analyse the qualitative data.

## Results

**Figure 1.**
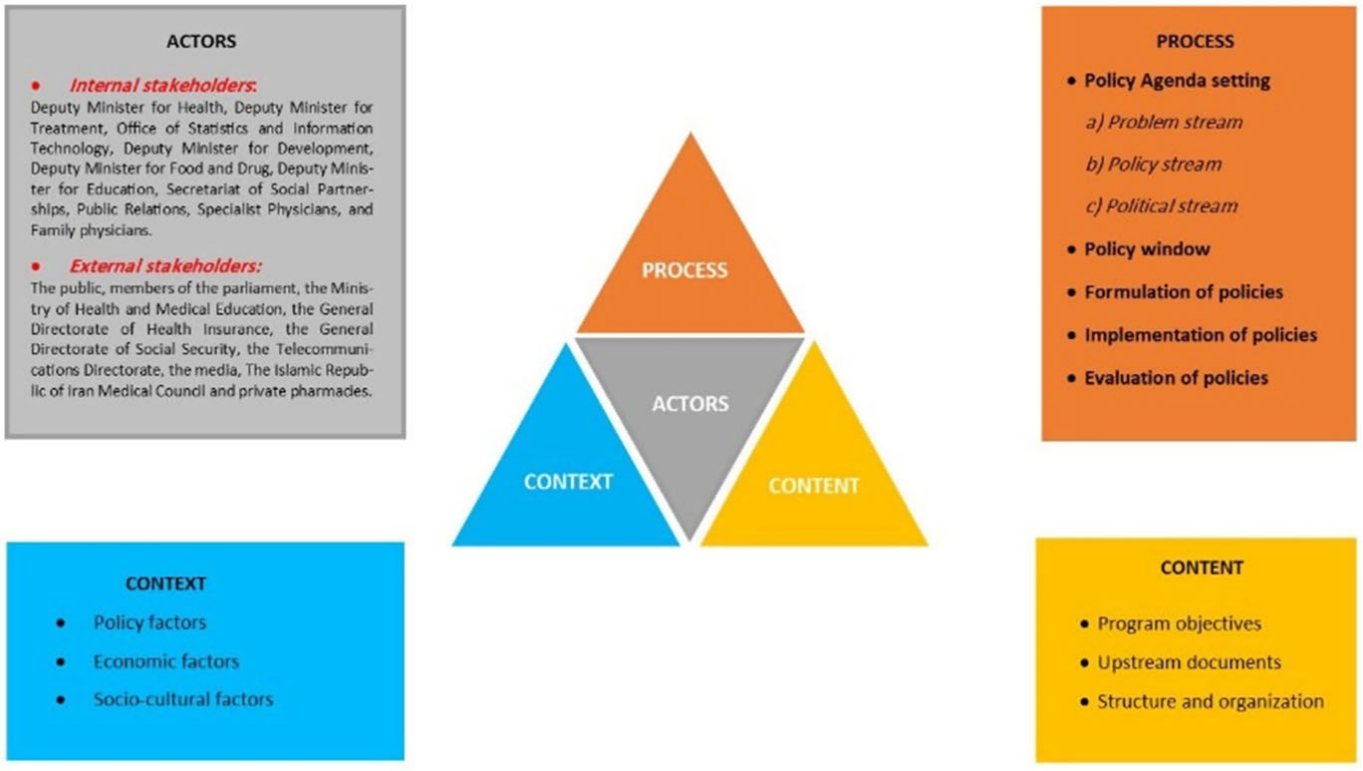
The policy framework of E-referral system in Iran based on Walt and Gilson’s triangle framework.

Results were reported based on four dimensions of the policy triangle model (context, content, process, and actors). The classification is explained in Figure 1.

### Context

The underlying factors affecting the e-Referral system included policy, economic, and sociocultural factors.

#### Policy factors

Comprehensive support was provided for the all-out establishment and implementation of the e-Referral system. The support was achieved via holding several meetings with government officials and members of the parliament. The pacing of implementation of the e-Referral system was affected by the replacement of managers and officials at the national level, so that the newly appointed managers were not fully aware of the importance and necessity of the programme.

*“A team was formed comprising the deputy of health and treatment, they visited all the cities of the province, held joint meetings with the council, the city governor, village governor, the provincial governor in the same way, and sought their support.” (an expert of the Deputy of Health of Golestan University of Medical Sciences).*

*“Since early 2018, in coincidence with the replacement of the Minister of Health and his deputies, we faced a great challenge. New managers were not in the picture. It took a long time to get fully aware of everything.” (Middle manager of Golestan University of Medical Sciences).*

#### Economic factors

Despite the limited resources required to implement the e-Referral system, adequate support was not provided at the national level to solve the financial problems. Delay in payment to family physicians and specialists has led to making the members of this treatment group less motivated. Despite the relatively low visit fee for specialists in the referral system, due to the high inflation rate, some patients could not afford to pay the fee.

*“Family physicians and specialists involved in implementing the project merely complained about financial issues and timely payment of claims, which has a direct relationship with the way of providing services to patients.” (an expert working with the Office of Deputy of Treatment of Golestan University of Medical Sciences).*

#### Sociocultural factors

According to the e-Referral process, for the patient in need of referral, in the first level, appointment is provided electronically to refer to the relevant specialist at the specified time and place. But despite making appointments for them, some patients do not visit to the designated physician. Some patients, based to their habits and as usual, tended to visit the specialists they know without a being visited by a family physician and to make an appointment to be referred to a specialist. The physician’s refusal to refer them to specialists on one hand and patients’ insistence on this on the other hand leads to growing tension between patients and the family physician. Also, many people were not fully aware of the process of the programme and the services provided.

*“Some people tend to directly visit a specialist and while asking the physician to provide them with a referral code, insist on their request and do not care about any instructions.” (an operational manager of Golestan University of Medical Sciences).*

*“We have not educated and informed the people as we had to. The e-Referral system is a change in the current process of providing services to the people. In case we tend to make a change, the necessary information and culture building must have been done prior to that.” (a senior manager, Golestan University of Medical Sciences).*

### Policy content

It consists of three sections: programme objectives, upstream documents, and structure and organization.

#### Programme objectives

From the experts’ point of view, the goals of the e-Referral system included ease of access, resources management, service continuity, fair use of services by the people, surrendering identity to the family physician, modification of people’s behaviour, and use of EHRs.

#### Upstream documents

Documents and laws developed for the implementation of the referral system at the national level, including the general policies of the health system communicated by the Supreme Leader, the laws of the fourth, fifth, and sixth development plans of the Islamic Republic of Iran, and the executive instructions of the first step referral system (villages and populations below 20,000 and nomads) (General policies of the system, 2006; The 5th 5-years Development Plan of Iran, 2015; The 6th 5-years Development Plan of Iran, 2017; Damari *et al.*, 2019).

#### Structure and organization

In order to work together to establish an e-Referral system in the university and its affiliated units, academic, technical, and support committees were formed. The main players of each team and the roles they should play in their capacity were identified. Natural and legal persons in this field were invited to participate in technical and specialized meetings, and all members were required to participate in the meetings and implement the programme.

### Policy process

The policy process includes policy agenda setting, policy formulation, policy implementation, and policy evaluation.

### Policy agenda setting

According to Kingdon’s model, the three streams – problem, policy, and political determination – need to come together to open the window of opportunity and put policy on the agenda.

#### a) Problem stream

Over the past years, a large number of specialist physicians have been trained and graduated in Iran, and patients’ tendency to refer directly to specialists has enhanced. Given the higher tariff of the specialists compared with that of the general practitioners, more people referring to specialists would lead to an increase in the payments by people and insurance organizations, which, due to lack of resources, will increase the burden of government spending. Prior to the deployment of the e-Referral system, no appointment was made for a certain specialist at an exact time. In these circumstances, there was a possibility of refraining from being visited by a specialist, cancellation of appointments, and absence of physician, which led to the patient’s dissatisfaction with the government system and his/her desire to attend the private sector intensified.

*“Many services could have been surrendered by a general practitioner, but with unnecessary visits to our specialists, the workload in specialized clinics would have increased a lot and the quality would have reduced accordingly.” (a middle manager of Golestan University of Medical Sciences).*

#### b) Policy stream

The then Minister of Health and Medical Education (2001–2005), at the time of receiving the vote of confidence from the parliament, promised to implement the family physician programme and referral system in the country. In 2004, the family physician policy was successfully included in the Fourth Five-Year Development Plan (2005–2009) (Ministry of Health & Medical Education, 2005) but the Ministry of Health and Medical Education (MOHME) could not allocate the required budget (Damari *et al.*, 2016). In March 2004, the annual budget for treatment in rural areas increased but this amount was spent on reducing the gap between villagers and did not allocate the fund to family physician and referral system but to equality of rendering services to the deprived people (Moshiri *et al.*, 2013). And since April 2005 it was implemented in rural-nomadic areas and cities with a population of less than 20,000 people (Health Policy Council, 2009). The then president announced that by March 2013, the programme of urban family physicians had to be implemented throughout the country. But in the end, only two provinces, Fars and Mazandaran, took the lead for its full implementation (Yazdani, 2011).

#### c) Political stream

The development of software and hardware infrastructures led to the creation of a suitable platform for deployment of e-Referral system. Health Information Software known as ‘NAB system’ was developed and launched by Golestan University of Medical Sciences. Through managing patient information, the system paved the way for mechanization of treatment and facilitated the patient referral process. According to the law passed by the Islamic Consultative Assembly, one percentage point per year is added to the value-added tax (VAT) rate of the government’s share as a health tax and, in coincidence with its receipt, it is directly deposited to the revenue line allocated for this purpose. Once the Act was passed, the resources needed to implement the referral system in the MOHME were considered. The strong and charismatic personality of the policymakers of the e-Referral system in the province led to the promotion of the programme and the involvement of stakeholders. The role, commitment, perseverance, and interest of the presenters and staff were effective factors in advancing the programme. The visits and studies carried out by the national officials on the progress of the implementation of the electronic system in the province, led to the persuasion and cooperation of the MOHME.

*“Golestan province had ‘Narmafzar Etellaate Behdashti (NAB) system’ that is independent of the country’s health information software. Golestan province had full authority on its own systems. In the ‘NAB system’, data was exchanged. Patients were referred to the hospital special clinic, but his/her information was sent to the Ministry, and then was resent to the special clinic. The cycle was established in Golestan province.” (A senior manager of Golestan University of Medical Sciences)*

### Policy window

According to the Kingdon’s theory, the three streams of problem, the stream of solution, and the stream of political determination must come together to open the window of opportunity and put a policy on the agenda of policymakers. If any of these issues have a problem, a policy is proposed but it is not put on the agenda. In the present study, the window of policy opened due to the confluence of the three above-mentioned streams.

### Formulation of policies

In order to develop e-Referral policies and programmes, the experiences of other countries such as Turkey, Thailand, the United Kingdom, and Canada were studied by an expert team. Then, the draft “Referral System Establishment Project” was compiled, and coordination meetings of the General Directorate of Health Insurance, Health Departments of Golestan University of Medical Sciences were held. During meetings and in the correspondence with the provincial governor and members of the Islamic Consultative Assembly, while informing them, their support was sought. The issue was explained in the meetings of the governorate’s working group on health and food security. Several meetings were held while the city and village officials were in attendance. Following these steps, Golestan University of Medical Sciences requested the MOHME to issue a permit to implement the e-Referral system. The MOHME approved and communicated the implementation of the programme after conducting several stages of review.

### Implementation of policies

E-Referral system programmes and policies were implemented using a bottom-up approach. The policy executors while having an important role to play in implementing the communicated policy and had a role in the policy-making process. Initially, the three cities of Aliabad Katoul, Aq Qala, and Bandar-e-Turkmen were selected as pilot cities for the implementation of the programme and gradually the project was extended to the whole province. In order to implement the e-Referral system, the required software, hardware, information, process, and manpower infrastructures were provided as much as possible. Through the ‘Behyab system (Integrated electronic patient guidance program)’, making electronic appointment became possible. Appointment kiosks were set up and through a mobile application people could make appointments. Of the total admission capacity of patients in specialized clinics, 50% was allocated to e-Referral patients. The patient was notified of the appointment cancellation through SMS. The patients were referred to specialists with a referral code, and following the visit and the required treatment measures, the specialists had to provide the relevant family physician with electronic feedback. In the case of any disruption in the Internet, a hard copy referral from a family physician to a specialist was performed.

### Evaluation of policies

Based on the checklists designed in the health care departments, periodic environmental monitoring was performed on the programmes of the e-Referral system of the health care networks. The management dashboard was used for monitoring applications. The information extracted from the e-Referral system dashboard includes the number and rate of referrals from level 1, and the number of patients for whom appointment was made from level 1. The number of patients referred to the relevant specialist according to the appointment made; the number and amount of feedback from level 2 to level 1 is the number and amount of referrals. The patients’ views were received through SMS surveys.

### Challenges of implementing programme

Challenges of implementing programme include appointments, referral load and waiting times in specialized clinics, visits, and feedback.

#### a) Appointment making

In some referral health centres, due to the plurality of their tasks they did not have enough time to make an appointment for the patient. In some cases, the referred patients were dissatisfied with the absence of a specialist at the appointment time, as well as the limited capacity of the number of patients to be visited by specialist.

*“The number of patients admitted to clinics, as well as the making appointments for some specialized fields is so low and patients are forced to visit specialists in the private offices.” (A family physician)*

#### b) Referral loads and waiting time in specialized clinics

The cancellation of the contract of the Health Service Insurance Organization with the private sector led to increasing the number of patients referring to the public sector, and in addition to the congestion of patients in specialized clinics, the waiting time became longer. In these circumstances, some specialists did not write back to family physicians.

*“Now that the health insurance organization no longer has a contract with the private sector, the number of referrals to clinics increases, and given the limited appointment making, the patient’s waiting time becomes longer. Therefore, the person must either accept to wait for a long time or pay the visit fee on his own.” (An expert of Vice Chancellor Office for Treatment, Golestan University of Medical Sciences)*

#### c) Visit and diagnosis

Under these conditions, the duration of visit time for each patient became shorter than the standard time and patients’ tendency towards visiting specialists in the private sector increased.

*“Although a standard time has been set for the visit, many of our specialists do not comply with this issue.” (A middle manager of Golestan University of Medical Sciences)*

*“Specialists are not required to provide quality services in state clinics similar to private clinics, which lowers down the quality of services in the public sector.” (A health network manager)*

#### d) Feedback of the specialists to the family doctors

Feedback written by a specialist was also of poor quality and in some cases could not be used by a family physician.

*“Family physicians say: due to various probable reasons specialists do not write a detailed report so that to be useful to us, as they are either not justified, or that they do not have time.” (A middle manager, Golestan University of Medical Sciences)*

### Actors

Actors fall into two categories: internal stakeholders and external stakeholders. Internal stakeholders (inside Golestan University of Medical Sciences) included Deputy Minister for Health, Deputy Minister for Treatment, Office of Statistics and Information Technology, Deputy Minister for Development, Deputy Minister for Food and Drug, Deputy Minister for Education, Secretariat of Social Partnerships, Public Relations, Specialist Physicians, and Family physicians. External stakeholders (outside Golestan University of Medical Sciences) included the public, members of the parliament, the MOHME, the General Directorate of Health Insurance, the General Directorate of Social Security, the Telecommunications Directorate, the media, the Islamic Republic of Iran Medical Council, and private pharmacies. Themes and sub-themes based on policy triangle and Kingdon’s models are indicated in Table 1.

**Table 1.** Themes and sub-themes based on policy triangle and Kingdon’s models

Theme	Sub-theme	Models
Context	Policy factors	Policy triangle
	Economic factors	
	Sociocultural factors	
Content	Programme objectives	Policy triangle
	Upstream documents	
	Structure and organization	
Process	Policy agenda setting	Kingdon
	Policy formulation	Policy triangle
	Policy implementation	
	Policy evaluation	
Actors	Internal stakeholders	Policy triangle
	External stakeholders	

## Discussion

Iran’s e-Referral system was launched as a pilot project in Golestan province with the aim of fair access and benefit to services and better management of health resources. A review of previous studies shows that a better and more effective referral system for pregnant mothers and children under 6 years of age leads to easy access to physicians and medicine for villagers, reduces treatment costs (Jannati *et al.*, 2010), and leads to reduction of costs (Kumar, 2016; Van Uden *et al.*, 2003). Although most of the available evidence indicate the effectiveness of the family physician and referral system in terms of health outcomes and improved accessibility, there is conflicting evidence in terms of controlling service consumption and the resulting economic burden. Thanh and Rapoport came to the conclusion that existence of a formal primary care physician increases services consumption (Thanh & Rapoport, 2017). Also, the analysis of data on the cost of health services in 18 member countries of the Organization for Economic Cooperation and Development (OECD) showed that the implementation of the family physician programme has not had a significant impact on reducing health costs (Delnoij *et al.*, 2000). Therefore, our analysis in this field should be conducted while taking into account environmental conditions such as the conditions of the health care system and service delivery system, social, economic, and cultural status of society and other factors impacting the receipt of services.

The implementation of e-Referral system in Iran is affected by political, economic, social, and cultural factors dominating the society. Implementing health programmes such as family physicians and referral systems suffers from constant changes in managers or policymakers. This factor either slows down the execution of programmes or makes them partially out of priority. This policy challenge has been mentioned in other studies (Nasiripour *et al.*, 2011; Roudpeyma *et al.*, 2003). In addition, the coincidence of implementation of this programme with sanctions has created significant threats to this programme and its expansion (Aeenparast *et al.*, 2017; Eskandari *et al.*, 2013). One of the major economic challenges was the delay in physicians’ financial payments. This factor leads to dissatisfaction or a negative impact on the performance of physicians (Ferdosi *et al.*, 2012). In addition, inadequate staff salaries have been mentioned in other studies (Sabatier, 2007; Nakhaee *et al.*, 2012). Inadequate public participation in adhering to the referral system was one of the most important cultural factors that could indicate lack of public awareness of the advantages of the referral system. Numerous studies have referred to the lack of public awareness of this programme as a challenge (Abbasi *et al.*, 2021; Safizadehe Chamokhtari *et al.*, 2018). Therefore, the first step in improving the attitude and behaviour of society is to enhance the public awareness and educating them (Etemad *et al.*, 2011). In this regard, the involvement of the mass media to achieve this goal is helpful.

The most important problems emanated from non-implementation of the referral system were weakening the position of general practitioners and patients not visiting them, the increase in the payment of people, and insurance organizations following the referral of more people to specialist doctors. Recent studies also confirm these problems (Eskandari *et al.*, 2013; Shiyani *et al.*, 2016). As a solution to the above problems, the deployment of family physician and referral system was proposed; however, despite the need to establish a referral system in the upstream documents and laws, the implementation of this programme was not welcomed. Factors such as conflict of interest, conflict between the ministries involved, and physicians’ lack of mastery of managerial knowledge and managerial instability have been some of the challenges in its formation (Nasiripour *et al.*, 2011).

Insufficient financial resources and insufficient required infrastructure were other reasons (Alaei *et al.*, 2021; Alipour *et al.*, 2019). The launch of the health information system provided the required platform to facilitate the patient’s e-Referral process. Following passing an Act on the allocation of 1% of value-added resources to villagers’ prevention and treatment plans within the framework of the referral system, hopes for financing the e-Referral system programme increased. In addition to this, the characteristics of policymakers and executives and the persuasive power of the country’s top managers led to the introduction of the e-Referral system. These characteristics of managers have also resulted in policy agenda in the study of Moshiri *et al.* (2016) and the study of Heidari *et al.* (2018). Therefore, with the temporary elimination of financial problems, the creation and expansion of electronic infrastructure, and the convening several meetings to resolve conflicts and also by holding training classes, a policy window was opened to put the e-Referral system on the agenda.

E-Referral system programmes and policies were implemented with a bottom-up approach. So that policy executors, in addition to implementing the programme, policymaking and adopting goals and selecting strategies to implement the policy, had a role in implementing it. The appointment making, visits load and waiting time in specialized clinics, diagnoses and feedback from a specialist to a family physician were the operational challenges in the implementation of the e-Referral system. These challenges have been emphasized in previous studies (Nasiripour *et al.*, 2011; Nakhaee *et al.*, 2012; Streubert Speziale *et al.*, 2011; Harper & Gamlin, 2003; Maftoon *et al.*, 2015; Farzadfar *et al.*, 2017; Torabi Ardakani *et al.*, 2015). Providing sustainable resources and allocating financial credits from the national level to the province, designing and implementing effective performance monitoring system, reforming the appointment system, and empowering medical students to play a role in the programme may play an effective role in improving the existing situation.

Family physicians and specialist physicians are among the most important players in the e-Referral system. Therefore, precise care should be applied to choosing knowledgeable, skilled, and capable family physicians as gatekeepers. Failure to provide feedback by some specialist physicians and problems in admitting referral patients and patients’ inclination towards referring to private clinics was another issue that could be attributed to the benefit of specialists and sub-specialists, disrespect for the competence and professionalism of general practitioners and poor supervision. Lack of cooperation on the part of specialists makes it difficult for family physicians to play their role (Abbasi *et al.*, 2021). People are one of the main beneficiaries of the e-Referral system. Inadequate facilities and services and lack of proper service of the first level is one of the reasons why the referral system is bypassed by the people. In order for the referral system to be properly implemented, the quality of service delivery must be improved, so that patients do not have to refer to higher levels for the treatment of a simple disease (Hosseini *et al.*, 2005).

### Strengths and limitations of the study

One of the strengths of the study was the use of a qualitative approach with two methods of in-depth individual interviews and document analysis. The two well-known conceptual frameworks of the policy analysis triangle and the Kingdon’s three-stream model used could collect data on process, content, context, and actors for policy analysis. However, this study had some limitations. This study was a cross-sectional study. Although attempts were made to consider maximum diversity in the samples, the samples were purposefully selected, which limits the possibility of generalization of the findings.

## Conclusions

The analysis of the policies of the e-Referral system helped to explain the agenda, and identify the components of the policymaking cycle and the political, economic, social, and cultural factors affecting it. It also helped to identify the stakeholders involved in the e-Referral system. In order for the programme to continue and succeed, creating the required determination and commitment on the part of ministers of health and medical education and senior managers of the health system in all governments, earmarking the resources and its sustainability for the programme by determining an independent line in the annual budget, improving allocation of resources through insurance management, reforming the payment system, planning to raise public awareness, and attracting community participation are essential for the support and acceptance needed in implementation is essential. In addition, utilizing the capacity of the media and social networks and non-governmental organizations for public culture is of importance.
